# Patient-reported outcomes and symptom clusters pattern of chemotherapy-induced toxicity in patients with early breast cancer

**DOI:** 10.1371/journal.pone.0298928

**Published:** 2024-02-23

**Authors:** Juan Adrian Wiranata, Susanna Hilda Hutajulu, Yufi Kartika Astari, Benedreky Leo, Bagas Suryo Bintoro, Mardiah Suci Hardianti, Kartika Widayati Taroeno-Hariadi, Johan Kurnianda, Ibnu Purwanto

**Affiliations:** 1 Clinical Epidemiology Study Program, Master of Clinical Medicine Postgraduate Program, Faculty of Medicine, Public Health and Nursing, Universitas Gadjah Mada, Yogyakarta, Indonesia; 2 Academic Hospital, Universitas Gadjah Mada, Yogyakarta, Indonesia; 3 Division of Hematology and Medical Oncology, Department of Internal Medicine, Faculty of Medicine, Public Health and Nursing, Universitas Gadjah Mada/Dr. Sardjito General Hospital, Yogyakarta, Indonesia; 4 Research Scholar, Division of Hematology and Medical Oncology, Department of Internal Medicine, Dr. Sardjito General Hospital, Yogyakarta, Indonesia; 5 Specialty Program in Internal Medicine, Department of Internal Medicine, Faculty of Medicine, Public Health and Nursing, Universitas Gadjah Mada, Yogyakarta, Indonesia; 6 Department of Health Behaviour, Environment, and Social Medicine, Faculty of Medicine, Public Health and Nursing, Universitas Gadjah Mada, Yogyakarta, Indonesia; 7 Center of Health Behaviour and Promotion, Faculty of Medicine, Public Health and Nursing, Universitas Gadjah Mada, Yogyakarta, Indonesia; University of Rome, ITALY

## Abstract

**Objective:**

This study aims to characterize patient-reported chemotherapy-induced toxicity in patients with breast cancer, determine its association with treatment regimens and patient characteristics, identify toxicity symptom clusters within a specific chemotherapy timeframe and analyze the correlation between symptom clusters within and between the timeframe to understand the changes and influences across chemotherapy.

**Methods:**

Forty-six patient-reported toxicities during neoadjuvant/adjuvant chemotherapy for breast cancer were evaluated using adapted CTCAE version 4.0. Chi-Square/Fisher’s Exact test was performed to analyze the difference in the incidence of toxicity symptoms by chemotherapy regimens. Poisson regression performed to assess factors associated with patient’s total chemotherapy toxicity. Exploratory factor analysis (EFA) conducted to identify symptom clusters at T1 (first half) and T2 (second half of planned cycle). Factor scores were generated and Spearman correlation performed to explore the factor scores correlation between symptom clusters.

**Results:**

A total of 142 patients with stage I-III breast cancer were included. The incidence of several toxicities differed significantly among three chemotherapy regimens. Subjects age ≥51 years are associated with lower number of reported toxicity (IRR/incidence rate ratio = 0.94, 95% confidence interval/CI 0.88 to 0.99, p = 0.042). Receiving more chemotherapy cycles are associated with higher number of reported toxicity (IRR = 1.06, 95% CI 1.03 to 1.10, p<0.001). Two symptom clusters identified at T1 (psychoneurological-pain/PNP-T1 and gastrointestinal-psychological/GIP-T1 cluster) and three at T2 (psychoneurological-pain/PNP-T2, epithelial/EPI-T2, and gastrointestinal cluster/GI-T2), with moderate-strong positive correlation between PNP-T1 and GIP-T2 (p<0.001), PNP-T1 and PNP-T2 (p<0.001), and GIP-T1 and PNP-T2 (p<0.001).

**Conclusions:**

This study investigated 46 patient-reported toxicities prospectively during adjuvant/neoadjuvant chemotherapy for early breast cancer. Anthracycline-taxane combination regimen had higher proportions of toxicity incidence. Subject’s age and number of chemotherapy cycles significantly associated with total number of toxicity symptoms. Two symptom clusters at T1 and three at T2 were identified, with significant correlation between symptom clusters within and between chemotherapy timeframe.

## Introduction

Breast cancer has the highest incidence in Indonesia. It accounts for 30.8% of all female cancers and contributes to 11% of cancer-related mortalities in 2020 [1]. The five-year survival rates for breast cancer in Indonesia are less favorable when compared to those in neighboring Asian countries [2], ranging from 51% for all stages to 12% for patients with late-stage disease [3, 4].

The use of chemotherapy in patients with breast cancer is proven to be efficacious and improve survival rates. However, chemotherapy also suppresses and damages normal cells and tissues, which can lead to toxicity symptoms [5]. Various toxicities associated with chemotherapy can have diverse impacts on the patient’s physical fitness, quality of life, and emotional state [6, 7]. Furthermore, they often cause clinicians to reduce dose intensity or postpone treatment in order to prevent or cope with the emergence of these toxicities. This situation, thereby, affects the relative dose intensity (RDI) [8, 9], and leads to decreased treatment efficacy and patient survival [10]. Accurate reporting and comprehension regarding chemotherapy-induced toxicity and its impact are essential to provide the basis for recommendations and decisions regarding its management.

The current understanding of the type and frequency of chemotherapy-induced toxicity remains oriented on reporting from clinical trials, which may not accurately reflect their occurrences within routine clinical settings. This disparity is often caused by the exclusion of individuals with a higher susceptibility to experiencing toxicity and complications in clinical trial settings, leading to the underrepresentation of these populations [11]. The toxicities highlighted in clinical trial publications tend to be selective and reported solely on the most prevalent or severe toxicities [12]. In addition, toxicity assessment in clinical trials predominantly relies on clinicians’ observation, which may not fully encompass the whole spectrum of occurrence and severity of the subjectively experienced toxicities by the patients [13]. To address this concern, the quantification and reporting of chemotherapy-induced toxicity in routine clinical practice have utilized patient-reported outcome measures, which have received growing attention in recent years [14]. This symptom-recording approach is still relatively understudied, particularly in patients with breast cancer.

In addition to identifying individual chemotherapy-induced toxicity symptoms, considering how these symptoms are interconnected is essential to enhance patient care delivery. Previous studies have shown that cancer patients can experience more than one, or even up to eight, distinct toxicities concurrently [15]. An approach to address this concern is to identify symptom clusters. A symptom cluster represents a set of symptoms that exhibit stability and independence and are strongly related to each other apart from their association with symptoms beyond the cluster boundaries. The results derived from the cluster analysis of chemotherapy-induced toxicity symptoms have become the basis for the groundwork of new strategies development aimed at managing toxicity symptoms simultaneously and enhance care delivery [16].

To date, many studies have investigated the symptom clusters of chemotherapy-induced toxicity in patients with breast cancer [17], but none have integrated symptom recording using CTCAE measures in a patient-reported manner. Report of chemotherapy toxicity using CTCAE in breast cancer chemotherapy itself is also limited. Existing studies also only examined few types of toxicities throughout chemotherapy [18, 19]. In Indonesia, no study has explored the incidence and symptom clusters of chemotherapy-induced toxicity in patients with breast cancer. Therefore, our study aims to characterize the profile of chemotherapy-induced toxicity using a patient-reported measure, determine the association of toxicities with treatment regimens, sociodemographic and clinicopathologic variables. We also aim to identify toxicity symptom clusters within a specific chemotherapy timeframe, and analyze the correlation between symptom clusters within and between the timeframe to understand the changes and influences across chemotherapy. The information provided in this study may give insight and additional knowledge to the existing literature regarding the profile and patterns of chemotherapy-induced toxicity and toxicity symptom clusters within our local population.

## Methods

### Study participants

The subjects were recruited prospectively from the Hematology and Medical Oncology Division, “Tulip”/Integrated Cancer Clinic, Dr. Sardjito General Hospital, Yogyakarta, Indonesia, between July 2018 and March 2022. The subjects included were women aged 18 years or older with histopathologically proven early (stage I-III) breast cancer, with Eastern Cooperative Oncology Group (ECOG) performance status of 0 and 1. All cases were scheduled for neoadjuvant or adjuvant first-line chemotherapy. Subjects with terminal illnesses and severe comorbidities were excluded. Written informed consent was obtained from each study participant. This study has received ethical approval from the joint ethics committee of the Faculty of Medicine, Public Health, and Nursing of Universitas Gadjah Mada/Dr. Sardjito General Hospital (reference number: KE/FK/0417/EC/2018).

Data regarding patient characteristics were obtained from the medical records and through interview at baseline. Sociodemographic data included age, education attainment, marital status, and occupation. The subject’s age was defined as the age at breast cancer diagnosis and further dichotomized based on the mean value into groups of patients with age less than or equal to 51 and with age greater than 51. For education attainment cases were categorized into two groups, those who completed a higher degree education and those with no formal education until completion of senior high school. Marital status was dichotomized into married and single or divorced or widowed. The occupation was defined as working status at the time of breast cancer diagnosis and categorized into individuals employed in formal sectors (encompassing administrative and professional jobs, and often situated in office environments, such as civil servants, educators, and private sector employees), individuals employed in informal sectors (jobs involving manual labor or a skilled trades, such as farmers, traders, freelancers, entrepreneurs), and individuals not actively engaged in employment (such as a housewife or retiree).

The clinicopathological data included body mass index (BMI), comorbidities, and clinical stage. BMI was defined based on WHO-Asia Pacific criteria and classified into underweight (<18.5 kg/m^2^), normal (18.5–22.9 kg/m^2^), overweight (23–24.9 kg/m^2^), and obese (≥25 kg/m^2^). Comorbidity was defined as the presence of any comorbid conditions. The clinical stage was defined based on the 7^th^ edition of the AJCC cancer staging system and dichotomized into stages I–II and III. Molecular subtypes were analyzed using immunohistochemical study and classified as luminal A, luminal B (either with HER-2 positive or HER-2 negative), HER-2 enriched, and triple-negative breast cancer (TNBC) [20]. Treatment data consisted of the type of surgery before chemotherapy and the chemotherapy regimen. The history of surgery prior to chemotherapy was dichotomized into biopsy/lumpectomy and mastectomy. Chemotherapy regimens were determined by treating oncologists and categorized into the anthracycline-taxane combination, anthracycline-based, and taxane-based regimens.

### Measures of patient-reported chemotherapy toxicities

A subset of chemotherapy-induced toxicity symptoms was assessed using a questionnaire developed based on descriptive terminology from the Common Terminology Criteria for Adverse Events (CTCAE) version 4 [21]. Two practicing general practitioners conducted translation into Bahasa Indonesia. A script containing a stepwise interview procedure and the questionnaire is also created to guide the interviewer during the interview. The developed questionnaire then underwent face validity evaluation, and pilot testing was conducted on ten lay persons with no medical field background from the research team acquaintance and reviewed by a medical oncologist, a cardiologist, and a neurologist.

One week after each cycle of chemotherapy and one week before the subsequent, face-to-face interviews with study subjects were conducted by a trained research team using the adapted CTCAE questionnaire. Per script, patients were first asked to report symptoms occurrence in the past week. For each symptom occurrence mentioned, details regarding the grade based on the CTCAE definition were further asked, with each definition explained by the interviewer. After all symptoms were mentioned, the interviewer was to ask about the occurrence and grade of all other symptoms not previously mentioned by the subject, following the list of symptoms in the script and first mentioning the definition of each symptom asked. All answers from the subject were recorded on the script’s checklist and then were later input into the database. Throughout the process, no clinician’s interpretation of the symptoms data was incorporated, and the interviewer only helped patients report their symptoms.

We encouraged patients to have a written note of any toxicity symptoms experienced during the week before the evaluation interview. Interviews were mainly done in the oncology outpatient clinic. At the designated meeting, the interviewer would record the toxicity symptoms narrated by the patients. Due to the COVID-19 pandemic situation, some interviews were conducted by telephone.

From the 80 symptoms in the questionnaire, we focused on 46 symptoms of chemotherapy side effects. These were selected *a priori*, based on what has been seen most reported in real-world studies and most frequently occurred in the clinical practice of breast cancer patients undergoing chemotherapy. These 46 chemotherapy-related toxicities were: dry mouth, oral mucositis, sore throat, dysphagia, dysgeusia, anorexia, nausea, vomiting, dyspepsia, flatulence, bloating, constipation, diarrhea, abdominal pain, dyspnea, cough, palpitation, back pain, paresthesia, chemotherapy-induced peripheral neuropathy (CIPN), myalgia, arthralgia, dry skin, pruritus, skin hyperpigmentation, hyperhidrosis, body odor, headache, dizziness, cognitive disturbance, memory impairment, insomnia, fatigue, anxiety, depression, alopecia, nail discoloration, nail ridges, watery eyes, blurred vision, tinnitus, irregular menstruation, vaginal discharge, vaginal dryness, decreased libido, and hot flushes. The toxicity grading for each chemotherapy toxicity is recorded according to the CTCAE version 4.0 definition [21].

### Statistical analysis

Data on subject characteristics and the incidence of 46 types of individual chemotherapy toxicity symptoms were presented as median, mean and standard deviation (SD) or as frequencies. Chi-Square/Fisher’s Exact test was performed to analyze the difference in the percentage incidence of each type of cumulative toxicity symptoms by chemotherapy regimen. The cumulative number of chemotherapy-induced toxicities experienced by each patient was determined by summing observations throughout their chemotherapy course, with a maximum possible toxicity count of 46 per patient. Each toxicity symptom was counted once per patient for each toxicity type, although patients may report the occurrence of the same type of toxicity during different cycles of chemotherapy. This approach is similar to the reporting technique commonly employed in clinical trials, which utilized CTCAE for symptom recording. Univariable Poisson regression analysis was then used to assess sociodemographic, clinicopathologic, and therapeutic variables associated with the patient’s total number of chemotherapy toxicity. Variables with a p-value of <0.250 were further analyzed using multivariable Poisson regression analysis, with a p-value of <0.05 deemed statistically significant.

Grouping individual chemotherapy-induced toxicity symptom events into symptom clusters was performed using exploratory factor analysis (EFA), which assumes symptoms in a cluster share common or latent factors, binding two or more symptoms together [22]. EFA analysis was conducted separately based on the time course of chemotherapy evaluation, including T1 (first half of planned chemotherapy cycle) and T2 (second half of planned chemotherapy cycle). The decision to dichotomize symptom cluster based on T1 and T2 are exploratory, based on a previous study which found that breast cancer patients may experience response shift to symptoms throughout their treatment course [23]. If the patients were planned to receive eight chemotherapy cycles, the first to fourth cycles would be determined as T1, and the fifth to eighth cycles would be determined as T2. If the patients were planned to receive six chemotherapy cycles, the first to third cycles would be determined as T1, and the fourth to sixth cycles would be determined as T2. Lastly, if the patients were planned to receive four chemotherapy cycles, the first and second cycles would be determined as T1, and the third and fourth cycles would be determined as T2. Each toxicity symptom was counted once per type of toxicity symptom per patient at each timeframe.

The Velicer’s minimum average partial (MAP) test [24] and parallel analysis [25] using the 95^th^ percentile of randomly generated eigenvalues were performed to determine the number of minimum and maximum possible factor solution extraction. All possible factor solutions between these values were analyzed. Tetrachoric correlations were used to create the matrix of associations for the dichotomous variables of toxicity symptoms occurrence. The EFA analysis was performed using the principal-component factor (PCF) extraction method and promax (oblique) rotation method. We used the PCF method over common factor analysis (FA) for several reasons. Given the extensive analysis of numerous toxicity types, our foremost consideration is data reduction, intending to focus on the minimum number of factor solutions required to capture the maximum proportion of the total variance present in the original set of variables. Additionally, common FA is susceptible to ’factor indeterminacy,’ wherein multiple sets of valid factor scores can exist for the same respondent. Since we conducted further data analysis on factor scores, PCA served as a pre-processing step for factor score generation [26].

For each factor solution model, individual items were moderated iteratively by excluding individual items with factor loading values less than 0.6 [26]. Items that had a factor loading value that met the cut-off point (>0.6) in two or more factor solutions (cross-loading) indicated that the individual item was not specific to a cluster and were excluded [27]. The reliability of the internal consistency of each toxicity symptom cluster was assessed using Cronbach’s Alpha. Items that proved to increase Cronbach’s Alpha significantly if removed were excluded, and clusters with a Cronbach’s Alpha value of >0.5 were retained [28].

The iteration of EFA was stopped when satisfactory quality was obtained, with at least three items loading on each factor deemed adequate [29]. The cumulative variance explained percentage of each factor solution was also evaluated, with the expected value of >50% [30]. The most applicable model from all possible factor solutions was decided by comparing each factor solution’s conceptual interpretability and meaningfulness. The determinant of the correlation matrix was assessed to check the likelihood of a multicollinearity problem, and the determinant greater than 0.00001 suggests that multicollinearity is not a problem [31]. Sample adequacy for the EFA analysis was tested using Kaiser-Meyer-Olkin (KMO) analysis to achieve a value above 0.6 as a requirement for adequate sampling and the significance of performing correlation matrix analysis. Bartlett’s test was performed, and significance was determined at p-value <0.05 to ensure adequate relationships within the set of variables [32].

Factor scores using the regression method were generated for each participant and each symptom cluster resulted from the selected EFA model. Using standardized score to a mean of zero, factor score resulted represents the values of the relative standing of each subject on the underlying factor, and a higher factor score indicated a higher standing on the scale and a better match to that factor, and vice versa [33]. Spearman correlation test was performed to explore the factor scores correlation of all factors produced by EFA at T1 and T2. A p-value <0.05 was considered statistically significant. EFA analysis, factor scores generation, Spearman correlation analysis and Poisson regression analysis were performed using STATA version 15 (Stata Corp, USA).

## Results

### Study participants

From 189 registered patients in this study, 35 were excluded due to not receiving chemotherapy and nine were excluded due to loss of follow-up. Additional three patients were excluded from the analysis because chemotherapy extended the observation period, hindering complete observation of toxicity outcomes. Finally, 142 patients were included for analysis (S1 Fig). The mean age of study participants is 51.7 years. The majority of the patients had an underweight BMI (<18.5 kg/m2; 37.3%), had at least one comorbidity condition (50.7%), were married (84.5%), had no formal education or had completed primary, junior, or senior high school (75.4%), and were either housewives or retired (47.2%). Most patients presented with stage III disease (57.7%), had a mastectomy procedure (85.2%), and received a combination regimen of anthracycline and taxane (77.4%) (Table 1).

**Table 1:** Study participants (n = 142) characteristics at breast cancer diagnosis.

Variables	Frequency (%)
Age	51.7 ± 8.4
≤51 years	67 (47.2)
>51 years	75 (52.8)
BMI (kg/m^2^)	24.3 ± 4.6
Underweight (<18.5)	53 (37.3)
Normal (18.5–22.9)	34 (23.9)
Overweight (23–24.9)	41 (28.9)
Obese (≥25)	14 (9.9)
Comorbidity	
Without comorbidity	70 (49.3)
With comorbidity(-ies)	72 (50.7)
Marital status	
Married	120 (84.5)
Single/widowed/divorced	22 (15.5)
Completed formal education	
Higher degree	35 (24.6)
No formal education to senior high school	107 (75.4)
Occupation	
Formal sector workers	37 (26.0)
Informal sector workers	38 (26.8)
Housewife/retired	67 (47.2)
Stage	
I-II	60 (42.3)
III	82 (57.7)
Molecular subtype	
TNBC	31 (21.8)
Luminal A	32 (22.6)
Luminal B	52 (36.6)
HER-2 positive	27 (19.0)
Surgery	
Biopsy/lumpectomy	21 (14.8)
Mastectomy	121 (85.2)
Chemotherapy regimen	
Anthracycline-taxane combination	110 (77.4)
Anthracycline-based	14 (9.9)
Taxane-based	18 (12.7)

Abbreviations: SD = standard deviation; BMI = body mass index; TNBC = triple negative breast cancer

### Incidence of chemotherapy toxicity symptoms and symptom distribution based on chemotherapy regimens

In total, 62.9% (4,107/6,532) of all reported toxicities are grade 1 toxicity, 8.7% (566/6,532) are grade 2 toxicity, and 0.4% (25/6,532) are grade 3 toxicity. No grade 4 toxicity was reported in this study (S1 Table). The ten most prevalent chemotherapy toxicity throughout the observation period were alopecia (99.3%), fatigue (98.6%), nail discoloration (98.6%), anorexia (97.9%), dysgeusia (97.2%), nausea (96.5%), nail ridges (93.7%), dry skin (92.3%), dry mouth (91.5%), and flatulence (90.8%). The toxicities of dry mouth (p = 0.043), oral mucositis (p<0.001), dysgeusia (p = 0.001), diarrhea (p<0.001), paresthesia (p = 0.024), dry skin (p = 0.023), skin hyperpigmentation (p = 0.004), body odor (p = 0.039), nail ridges (p = 0.021), and watery eyes (p = 0.024) are found to be significantly different between three chemotherapy regimens throughout the observation period (Table 2).

**Table 2:** Incidence of patient-reported chemotherapy toxicity symptoms by regimen throughout chemotherapy.

Toxicities	All regimen (%)	Anthra-taxane combination (n = 110) (%)	Anthracycline-based (n = 14) (%)	Taxane-based (n = 18) (%)	p
Dry mouth	91.5	94.5	78.6	83.3	0.043*
Oral mucositis	72.5	80.9	50.0	38.9	<0.001*
Sore throat	33.1	37.3	21.4	16.7	0.158
Dysphagia	66.2	70.0	42.9	61.1	0.131
Dysgeusia	97.2	100.0	78.6	94.4	0.001*
Anorexia	97.9	98.2	100.0	94.4	0.538
Nausea	96.5	97.3	100.0	88.9	0.193
Vomiting	76.8	77.3	71.4	77.8	0.886
Dyspepsia	74.7	76.4	64.3	72.2	0.523
Flatulence	90.8	90.9	85.7	94.4	0.761
Bloating	73.2	75.5	64.3	66.7	0.479
Constipation	80.3	81.8	78.6	72.2	0.576
Diarrhea	58.5	60.0	14.3	83.3	<0.001*
Abdominal pain	63.4	62.7	50.0	77.8	0.282
Dyspnea	42.3	42.7	28.6	50.0	0.506
Cough	74.6	77.3	64.3	66.7	0.386
Palpitation	49.3	50.9	50.0	38.9	0.669
Back pain	84.5	87.3	78.6	72.2	0.172
Paresthesia	76.1	80.9	50.0	66.7	0.024*
CIPN	65.5	66.4	50.0	72.2	0.371
Myalgia	83.8	84.5	71.4	88.9	0.402
Arthralgia	68.3	71.8	57.1	55.6	0.246
Dry skin	92.3	95.5	78.6	83.3	0.023*
Pruritus	78.9	80.0	57.1	88.9	0.096
Skin hyperpigmentation	90.1	94.5	78.6	72.2	0.004*
Hyperhidrosis	52.1	50.9	50.0	61.1	0.703
Body odor	59.9	62.7	28.6	66.7	0.039*
Headache	87.3	86.4	85.7	94.4	0.673
Dizziness	90.1	89.1	92.9	94.4	0.886
Cognitive disturbance	55.6	58.2	50.0	44.4	0.510
Memory impairment	60.6	63.6	50.0	50.0	0.428
Insomnia	79.6	80.9	64.3	83.3	0.331
Fatigue	98.6	98.2	100.0	100.0	1.000
Anxiety	68.3	70.0	57.1	66.7	0.638
Depression	64.8	64.5	64.3	66.7	1.000
Alopecia	99.3	99.1	100.0	100.0	1.000
Nail discoloration	98.6	99.1	100.0	94.4	0.401
Nail ridges	93.7	96.4	78.6	88.9	0.021*
Watery eyes	70.4	75.5	42.9	61.1	0.024*
Blurred vision	64.8	66.4	42.9	72.2	0.179
Tinnitus	51.4	51.8	50.0	50.0	1.000
Irregular menstruation	45.1	46.4	28.6	50.0	0.443
Vaginal discharge	40.8	40.0	42.9	44.4	0.956
Vaginal dryness	43.0	44.5	28.6	44.4	0.581
Decreased libido	45.1	46.4	35.7	44.4	0.764
Hot flushes	61.3	65.5	42.9	50.0	0.170

Abbreviations: CIPN = chemotherapy-induced peripheral neuropathy.

* = p <0.05

### Factors associated with total number of chemotherapy toxicity symptoms

In the multivariable analysis, we found that patients with age greater than or equal to 51 years are associated with a lower number of reported toxicity types (IRR/incidence rate ratio = 0.94, 95% confidence interval/CI 0.88 to 0.99, p = 0.042). Receiving a greater number of chemotherapy cycles are associated with a higher number of reported toxicity types (IRR = 1.06, 95% CI 1.03 to 1.10, p<0.001) (Table 3).

**Table 3:** Sociodemographic and clinicopathologic factors associated with total number of chemotherapy toxicity types.

Variables	IRR (95% CI)	P	Adjusted IRR (95% CI)	P
Age (years)				
≤51 years	Ref		Ref	
>51 years	0.92 (0.86–0.98)	0.015	0.94 (0.88–0.99)	0.042*
Marital status				
Married	Ref		Ref	
Single/widowed/divorced	0.89 (0.80–0.99)	0.048	0.90 (0.80–1.01)	0.066
Education attainment				
Higher degree	Ref			
No formal education to senior high school	0.98 (0.91–1.05)	0.580		
Occupation				
Formal sector workers	Ref			
Informal sector workers	1.01 (0.92–1.11)	0.867		
Housewife/retired	1.02 (0.93–1.11)	0.681		
BMI (kg/m^2^)				
Obese (<18.5)	Ref			
Overweight (18.5–22.9)	0.97 (0.89–1.06)	0.552		
Normal (23–24.9)	1.03 (0.94–1.12)	0.517		
Underweight (≥25)	0.98 (0.86–1.12)	0.798		
Comorbidity				
Without comorbidity	Ref			
With comorbidity(-ies)	1.00 (0.94–1.07)	0.925		
Stage				
I-II	Ref			
III	1.01 (0.94–1.09)	0.755		
Molecular subtype				
TNBC	Ref		Ref	
Luminal A	0.94 (0.85–1.04)	0.262	0.95 (0.86–1.05)	0.303
Luminal B	1.07 (0.99–1.16)	0.086	1.07 (0.99–1.15)	0.079
HER-2 positive	1.02 (0.92–1.14)	0.682	1.05 (0.95–1.16)	0.296
Surgery				
Biopsy/lumpectomy	Ref			
Mastectomy	0.97 (0.88–1.08)	0.612		
Chemotherapy regimen				
Taxane-containing	Ref		Ref	
Anthracycline-containing	0.87 (0.73–1.04)	0.129	0.94 (0.79–1.13)	0.506
Anthracycline-taxane combination	1.06 (0.97–1.15)	0.216	0.94 (0.84–1.06)	0.310
Number of cycle(s)	1.06 (1.03–1.08)	<0.001	1.06 (1.03–1.10)	<0.001*

Abbreviations: IRR = incidence rate ratio; CI = confidence interval; BMI = body mass index; Ref = reference; TNBC = triple negative breast cancer

* = statistically significant

### Exploratory factor analysis

At T1, MAP analysis reveals that a one-factor solution should be extracted, with a minimum average squared partial correlation value of 0.014. The parallel analysis reveals that nine factors solution should be extracted with eigenvalues greater than the randomly generated data (S2 Fig). All possibilities of factor solutions between one and nine were examined. At T2, MAP analysis reveals that a three-factor solution should be extracted with a minimum average squared partial correlation value of 0.015. The parallel analysis reveals that seven factors solution should be extracted with eigenvalues greater than the randomly generated data (S3 Fig). All possibilities of factor solutions between three and seven were examined.

The results of the selected EFA model for chemotherapy toxicity in T1 (two-factor model) and T2 (three-factor model) were selected and shown in Table 4. At T1, the determinant of the correlation matrix results was 0.056, suggesting no multicollinearity problem. The KMO measure of sampling adequacy results was 0.735, indicating the sampling adequacy to conduct EFA, with significant the Bartlett test of sphericity measure (p<0.001). The analysis yielded two symptom clusters with 13 items of toxicity symptoms. The first symptom cluster was termed the psychoneurological-pain symptom cluster, which contains toxicity items of memory impairment, alopecia, back pain, cognitive disturbance, paresthesia, insomnia, depression, and myalgia. This symptom cluster contributed 34.4% of the variance, with an Eigenvalue of 4.67 and a Cronbach’s Alpha of 0.71. The second symptom cluster was termed the gastrointestinal-psychological symptom cluster, which contains toxicity items of anorexia, nausea, dry skin, bloating, and anxiety. This symptom cluster contributed 30.6% of the variance, with an Eigenvalue of 3.62 and a Cronbach’s Alpha of 0.66.

**Table 4:** Exploratory factor analysis of chemotherapy general toxicity symptom cluster at T1 and T2.

Time	Cluster Name	Item	Factor Loading ^(a)^		Variance (%)	Eigenvalue	Cronbach’s Alpha
**T1**	Psychoneuro-logical-Pain Cluster	Memory impairment	0.833		34.4	4.67	0.71
		Alopecia	0.821				
		Back pain	0.772				
		Cognitive disturbance	0.759				
		Paresthesia	0.698				
		Insomnia	0.680				
		Depression	0.662				
		Myalgia	0.658				
	GI-Psychological Cluster	Anorexia	0.908		30.6	3.62	0.66
		Nausea	0.874				
		Dry skin	0.788				
		Bloating	0.775				
		Anxiety	0.715				
	**Determinant of the correlation matrix:**			0.056			
	**KMO Measure of Sampling Adequacy:**			0.735			
	**Bartlett Test of Sphericity:**			X^2^ = 391.9; df = 78; p = <0.001			
**T2**	Psychoneuro-logical-Pain Cluster	Memory impairment	0.936		31.1	4.77	0.74
		Cognitive disturbance	0.887				
		Fatigue	0.800				
		Depression	0.763				
		Anxiety	0.686				
		Myalgia	0.672				
	Epithelial Cluster	Dysgeusia	0.974		28.8	2.99	0.68
		Oral mucositis	0.901				
		Dry mouth	0.847				
		Dysphagia	0.808				
	GI Cluster	Vomiting	0.993		22.5	2.17	0.65
		Nausea	0.911				
		Anorexia	0.718				
	**Determinant of the correlation matrix:**			0.032			
	**KMO Measure of Sampling Adequacy:**			0.666			
	**Bartlett Test of Sphericity:**			X^2^ = 465.6; df = 78; p = <0.001			

^(a)^ Extraction method used = principle component factor, rotation method = promax rotation

^(+)^ Factor loading shown here with factor loading >0.6, and does not have cross-loading with other factors extracted

Abbreviations: T1 = time 1, first-half of chemotherapy cycle; T2 = time 2, second-half of chemotherapy cycle; GI = gastrointestinal; KMO = Kaiser-Meyer-Olkin; X^2^ = chi-square; df = degree of freedom.

At T2, the determinant of the correlation matrix results was 0.032, suggesting no multicollinearity problem. The KMO measure of sampling adequacy result was 0.666, indicating the sampling adequacy to conduct EFA, with significant the Bartlett test of sphericity measure (p<0.001). The analysis yielded three symptom clusters with 13 items of toxicity symptoms. The first symptom cluster was termed the psychoneurological-pain symptom cluster, which contains toxicity items of memory impairment, cognitive disturbance, fatigue, depression, anxiety, and myalgia. This symptom cluster contributed 31.1% of the variance, with an Eigenvalue of 4.77 and a Cronbach’s Alpha of 0.74. The second symptom cluster was termed the epithelial symptom cluster, which contains toxicity items of dysgeusia, oral mucositis, dry mouth, and dysphagia. This symptom cluster contributed 28.8% of the variance, with an Eigenvalue of 2.99 and a Cronbach’s Alpha of 0.68. Lastly, the third symptom cluster was termed the gastrointestinal symptom cluster, which contains toxicity items of vomiting, nausea, and anorexia. This symptom cluster contributed 22.5% of the variance, with an Eigenvalue of 2.17 and a Cronbach’s Alpha of 0.65. The detailed patterns of incidence and severity of these chemotherapy toxicities incorporated in these clusters at T1 and T2 throughout the study observation period were also provided (S4 Fig).

### Correlation between factor scores of chemotherapy-induced toxicity symptom clusters

The results of factor score extraction from symptom clusters resulted from the EFA, and the correlation of factor scores between each symptom cluster is shown in Table 5. We found that the psychoneurological-pain cluster at T1 has a significant positive strong correlation with the gastrointestinal-psychological cluster at T1 (r = 0.817; p<0.001), a positive moderate correlation with the psychoneurological-pain cluster at T2 (r = 0.579; p<0.001) and a positive weak correlation with the epithelial cluster at T2 (r = 0.258; p = 0.002). The gastrointestinal-psychological cluster at T1 has a significant positive moderate correlation with the psychoneurological-pain cluster at T2 (r = 0.609; p<0.001) and a positive weak correlation with the epithelial cluster at T2 (r = 0.260; p = 0.002).

**Table 5:** The factor scores correlation of symptom clusters at T1 and T2.

Variables	Factor Score Median (Min, Max)	PNP-T1	GIP-T1	PNP-T2	EPI-T2	GI-T2
Psychoneurological-Pain Cluster at T1	1.645 (0.890, 2.046)	1.00				
GI-Psychological Cluster at T2	0.106 (–0.710, 0.571)	r = 0.817 **p<0.001**	1.00			
Psychoneurological-Pain Cluster at T2	0.796 (0, 1.325)	r = 0.579 **p<0.001**	r = 0.609 **p<0.001**	1.00		
Epithelial Cluster at T2	0.780 (–0.145, 1.384)	r = 0.258 **p = 0.00**	r = 0.260 **p = 0.002**	r = 0.153 p = 0.070	1.00	
GI Cluster at T2	0.785 (–0.699, 1.342)	r = 0.004 p = 0.960	r = 0.019 p = 0.820	r = 0.150 p = 0.074	r = –0.100 p = 0.235	1.00

Abbreviations: GI = gastrointestinal; PNP-T1 = Psychoneurological-Pain Cluster at T1; GIP-T1 = Gastrointestinal-Psychological Cluster at T1; PNP-T2 = Psychoneurological-Pain Cluster at T2; EPI-T2 = Epithelial Cluster at T2; GI-T2 = Gastrointestinal Cluster at T2

## Discussion

This study is the first in Indonesia to investigate patient-reported toxicities recorded prospectively during adjuvant and neoadjuvant chemotherapy program for breast cancer in a real-world setting. The results of this study may give insight to policymakers for advocating for the implementation and sustainable development of symptom-recording systems reliant on patient-reported outcomes, a process that demands substantial resources. The profile of patient-reported toxicity and symptom cluster in this study might give an insight into the pattern of toxicity of local patients with breast cancer.

Recording symptoms using patient-reported outcome measures offers an opportunity for patients to describe their symptoms, which fosters patient engagement in tracking changes to their own well-being. This approach also promotes patient adherence to treatment protocols and enhances the overall treatment experience. Adopting patient-reported toxicity symptoms recording can mitigate potential observer bias that could arise from clinicians, which tend to emphasize symptoms they consider most relevant or frequent. Within time-constrained clinician-patient interactions, this method can also promote the effectiveness and efficiency of symptom communication [34].

Although a companion tool for CTCAE, known as the Patient-Reported Outcomes version of the Common Terminology Criteria for Adverse Events (PRO-CTCAE) has been created to assess symptomatic toxicities through self-reporting [35], there are several difficulties in implementing this approach that leads us to a novel approach by developing a patient-reported version of the CTCAE questionnaire and the interview process. First, certain symptom items in PRO-CTCAE have a dual assessment for severity and interference, and there are currently no standardized scoring rules for merging these attributes into a unified score in a single symptom. Moreover, ideally, the PRO-CTCAE questionnaire is implemented through a web-based software platform or a telephone interactive voice response system, allowing patients to report their symptoms independently. However, in our study population, many of our patients were not technologically illiterate, and we currently lack the resources to facilitate electronic questionnaire administration. Despite providing patients with a paper form of the questionnaire to take home and complete, the diverse educational backgrounds of the patients posed challenges in effectively filling out the questionnaire.

Consistent with observational studies that examined chemotherapy toxicity symptoms in breast cancer patients using patient-reported outcome measures [18, 19, 36], we reported a higher incidence of toxicity compared to what is reported in clinician-reported clinical trials setting. However, our study results also revealed a significant grade 1 chemotherapy toxicities proportion, with comparatively limited grade 2 and 3 toxicities and no grade 4 general toxicity occurrences. The incidence of grade 2 to 4 toxicities in this study is lower than in the aforementioned observational studies [18, 19]. The inclusion of only patients with good performance may contribute to the observed lower reporting of moderate to severe general toxicity symptoms in our study compared to others that included patients with low physical status. Other study also observed that individuals with poorer performance status (ECOG PS 2 to 4) reported symptoms with greater severity than patients with better performance status (ECOG PS 0 and 1) [37].

Ethnicity differences could also contribute to variation in response to a drug and toxicity rates, which encompasses genetic factors (ethnic-specific receptor mutations and gene polymorphisms) and population-level elements (environment, lifestyle, health-seeking behavior, and health policies) [38]. A prior study has observed that patients with breast and lung cancer of Asian ethnicity had a higher rate of severe neutropenia compared to Caucasian ethnicity [39]. Further exploration regarding the role of ethnicity in the incidence of non-hematological chemotherapy-induced toxicity can contribute to providing information on this phenomenon.

Cultural backgrounds might also impact symptom reporting. The perception of the body’s physiological state, known as interoception, forms the foundation for subjective emotions and feelings. Interoceptive awareness relates to the identification and determining the frequency and significance of bodily sensations and is a top-down process driven by concern, belief, and hope. Interoceptive accuracy involves accurately inferring the cause and degree of body change by processing the detection of cues from the body and its physiological features, which is a bottom-up process. Prior empirical and ethnographic studies show that the Asian population exhibit higher interoceptive awareness than the Western population while exhibiting lower interoceptive accuracy due to reliance on contextual cues, thus affecting the degree of symptom reporting [40]. Beyond cultural factors, spirituality and religiosity might also contribute to the symptom severity reporting. A prior study has observed that higher spirituality was associated with reduced severity degree of certain chemotherapy toxicity symptoms recorded in a patient-reported manner [41]. Previous survey has also observed that the majority of the Indonesian population attribute the significance of religion in their life [42]. Asian ethnicity, interoceptive traits and religiosity within the present study population may contribute to the low reporting of moderate to severe toxicity symptoms, and warrant further investigations.

We presented the data on the incidence of 46 types of chemotherapy-induced toxicity, which are greater compared to previous reports that utilize the same instrument. The description of this wide range of toxicity can give healthcare providers an insight into a broad range of toxicity that patients with breast cancer undergoing chemotherapy might experience. Some toxicities reported in this study, which may not pose an immediate life-threatening risk, are often missed by caregivers and are understudied. Symptoms such as skin hyperpigmentation, nail discoloration and ridges, which are cosmetically unpleasing and distressing, and symptoms such as tinnitus, watery eyes, and blurred vision, have substantial incidence in this study. However, they are often overlooked in clinical practice [43–45]. The diverse impact of chemotherapy highlights the significance of multidisciplinary approaches in organizing care complexity brought by chemotherapy toxicity. Addressing these can increase overall treatment experience and quality of life and allow patients to receive appropriate and timely management for specific toxicities [45, 46].

Our analysis found that anthracycline-taxane combination regimens are observed to have predominated the higher proportions of individual toxicities compared to the other regimen. Being a longer duration regimen with a greater number of agents, it is expected that anthracycline-taxane combination regimens may cause the emergence of more types of toxicity, which warrants clinicians to be aware of [36].

In our analysis, we found that older patients reported fewer toxicity symptoms, in line with a previous study in various solid tumors [47]. This is possibly due to an age-related psychological shift, reflecting an alteration in an individual’s internal framework for evaluating experienced symptoms, shaped by their lifetime exposure to symptoms or their encounter with symptoms from other medical conditions [48]. The pattern difference of toxicity symptoms reported in these populations warrants further studies and underlines that more attention is needed when evaluating toxicity symptoms in those populations. We also observed that patients who received a greater number of chemotherapy cycles report more toxicity symptoms, which is expected since longer exposure to chemotherapy may result in more emergence of toxicity symptoms, as also demonstrated in prior study [36].

Our cluster analysis found a psychoneurological-pain cluster at T1 (PNP-T1), gastrointestinal-psychological cluster at T1 (GIP-T1), psychoneurological-pain cluster at T2 (PNP-T2), epithelial cluster at T2 (EPI-T2), and gastrointestinal cluster at T2 (GI-T2). Although individual toxicity symptoms might vary within the cluster, gastrointestinal and psychoneurological symptom clusters are among the most widely established in symptom cluster studies of breast cancer patients undergoing chemotherapy [49], and addressed in clinical trials and cancer care [16]. The variation may stem from variance in symptom recording measures, treatment, and population across studies.

The clustering of symptoms could occur due to shared underlying etiology, the emergence of symptoms triggered by other symptoms, and side effects of symptomatic treatment that induce additional symptoms [50]. In PNP-T1 and PNP-T2, symptoms of anxiety, depression, insomnia, cognitive disturbance and memory impairment can potentially emerge from inflammation and structural changes within the central nervous system induced by chemotherapy [51, 52]. The interrelation between pain and psychological symptoms also have been well recognized, involving various biological, psychological, and social factors [53]. In PNP-T1, clustering of alopecia with psychological symptoms may also highlight the distress and impact on psychosocial well-being caused by the occurrence of alopecia [54]. Clustering within gastrointestinal symptom cluster in GIP-T1 and GI-T2, and within epithelial symptom cluster in EPI-T2, highlights the systemic nature of chemotherapy impact on rapidly dividing cells [55]. In GIP-T2, anxiety has the potential to influence the emergence of anticipatory nausea and vomiting, thereby contributing to the onset of chemotherapy-induced nausea or vomiting [56].

The symptom cluster’s factor score, which reflects the standing of an individual within each symptom cluster, exhibited a significant correlation both within and between the chemotherapy timeframe (T1 and T2). The findings of a positive correlation within the same timeframe between PNP-T1 and GIP-T1 suggests that gastrointestinal symptoms might cause emotional distress and decreased social functioning, which may drive the psychoneurological cluster symptoms [57, 58]. The positive correlation within a timeframe might also be caused by the influence of similar underlying factors, mechanisms, or physiological processes at the same timeframe [16].

Positive correlation between symptom cluster’s factor scores at different timeframes indicated that symptom cluster at a timeframe somehow related to or predictive of the symptom patterns in the other timeframe. The persistence of anxiety, depression, cognitive disturbance and memory impairment throughout chemotherapy might also drive the positive correlation of PNP-T1 and PNP-T2 [52, 59]. The positive correlation between the factor scores of GIP-T1 and the factor score of the PNP-T2 might reflect distress resulting from the negative anticipation of recurrent toxicities within the GIP-T1 symptom cluster [60]. The weak correlation between PNP-T1 and EPI-T2, and GIP-T1 and EPI-T2, suggests that there is only a minimal relationship between the two clusters, which may not have practical implications. Further studies are warranted to explore this between symptom clusters correlation.

The crosstalk between individual toxicity in a symptom cluster and between symptom clusters underlines the importance of treating individual symptoms and symptom clusters. Understanding the nature of the relationships among clustered symptoms and between symptom clusters is essential for tailoring treatment strategies. Knowledge of symptom clusters also gives healthcare providers insight into communicating more comprehensively with patients so patients can understand the potential range of symptoms they might experience concurrently, helping them prepare and manage their expectations.

Previous intervention studies have highlighted the positive outcomes of various complementary interventions that might manage symptom clusters. Various interventions, such as brief cognitive-behavioral strategies [61] and auricular point acupressure [62], have been applied for the pain, fatigue, and insomnia symptom cluster. A 16-week dance therapy [63], Tai Chi [64], and somatic acupressure [65] have been used for fatigue, insomnia, and depression symptom. The positive outcomes from the above intervention studies highlight the importance of addressing patients’ physical fitness and psychological well-being, which, through various complementary interventions, can simultaneously improve both physical and psychological outcomes.

Preliminary cluster analysis can provide a strong basis for establishing a symptom cluster as an outcome of specific cluster interventions. Prior studies conducted symptom cluster analysis of patient-reported toxicity and implemented a 16-week supervised exercise intervention during adjuvant chemotherapy to evaluate the changes in symptom clusters. Three symptom clusters were identified from the cluster analysis, including the ’emotional’, ’treatment-related’, and ’physical’ symptoms clusters. After completing the treatment, patients in the physical exercise groups reported lower symptom burden scores for the emotional symptom cluster compared to usual care [66]. The results of this study can give insight into determining intervention studies endpoint to address clustered toxicity symptoms simultaneously.

The strength of our study covers the prospective recording of symptoms with a sufficient follow-up period, which provided a comprehensive insight into the pattern of chemotherapy toxicity. Having a trained interviewer present during the data collection process offers aid for subjects from diverse economic, social, and cultural backgrounds in describing their symptoms, as opposed to using a checklist for self-reporting.

The present study has some limitations. This study is a single-center study with an observational design and consecutive sampling method, which may inevitably introduce selection bias and require a careful generalization of our findings. Although the questionnaire adapted from CTCAE has undergone face validation and pilot testing, it still needs further psychometric properties evaluation. Recording toxicity symptoms with weekly scheduled visits in this study might still have inevitably introduced recall bias. We have mitigated this by encouraging patients to keep notes about the symptoms. Currently, we are unable to implement an electronically integrated recording system that enables symptom reporting in real-time due to constraints in patients’ ability to adapt to this system. Another limitation is that we have yet to record symptomatic treatment for individual toxicity that might also affect symptoms by triggering or lowering symptoms recorded in this study.

In the analysis, another limitation is that the utilization of the PCA method in EFA may encompass only a small portion of individual-specific and unique variance (e.g., chemotherapy regimens and age). This is because the primary objective of the analysis is to uncover general patterns and facilitate data reduction within our local population rather than revealing specific latent factors, as is the case in the common FA method. Secondly, we have yet to address the problem of multiple comparisons in the analyses, which might give rise to false positive significant findings. As our study is exploratory, future studies employing confirmatory analyses with stringent multiple comparison controls and validating the symptom cluster models are warranted.

## Conclusions

The local patients with early breast cancer undergoing adjuvant/neoadjuvant chemotherapy experience a wide range of chemotherapy-induced toxicity symptoms. These toxicity symptoms are interconnected, either influencing or triggering one another. Understanding the interconnection between these toxicity symptoms into a symptom cluster offers valuable insights for developing interventions that can simultaneously address multiple symptoms, which can reduce resources and treatment complexity. The interconnection between toxicity symptoms also underlines the importance of adopting a multidisciplinary approach to managing toxicity symptoms to enhance patient treatment experience, quality of life, and health outcomes. The data on chemotherapy-induced toxicity of local patients might provide insight to health authorities and policymakers in improving care delivery for patients with breast cancer.

## Supporting information

S1 Fig Diagram for inclusion and exclusion of study participants. (TIF)

S2 Fig Parallel analysis scree plot using 95^th^ percentile of randomly generated eigenvalues of the chemotherapy toxicity symptom cluster at T1. (TIF)

S3 Fig Parallel analysis scree plot using 95^th^ percentile of randomly generated eigenvalues of the chemotherapy toxicity symptom cluster at T2. (TIF)

S4 Fig The incidence and severity pattern of chemotherapy toxicity symptoms incorporated in the symptom clusters throughout study observation period. (TIF)

S1 Table Incidence of patient-reported chemotherapy toxicity symptoms based on severity grade throughout chemotherapy. (DOCX)

S1 Dataset (XLS)
