# Supplementary figures and images for: Patient-reported outcomes and symptom clusters pattern of chemotherapy-induced toxicity in patients with early breast cancer
Source: PLoS One. 2024 Feb 23;19(2):e0298928. doi: 10.1371/journal.pone.0298928 (PMC10890761; doi:10.1371/journal.pone.0298928)

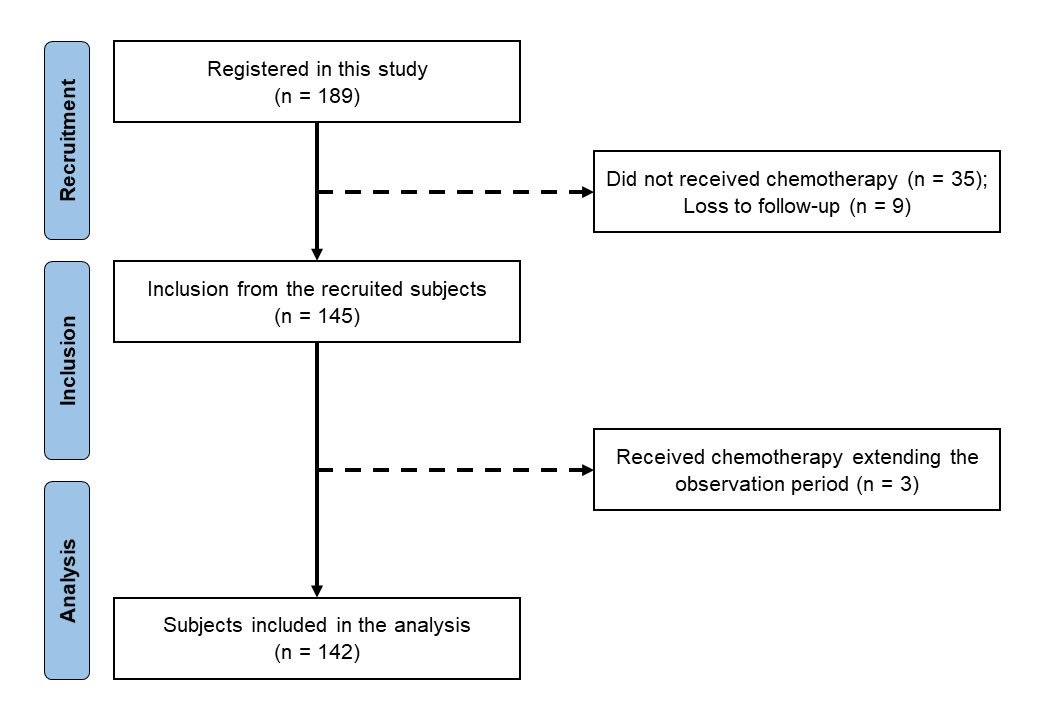

Supplement: S1 Fig — (TIF) [file pone.0298928.s001.tif]

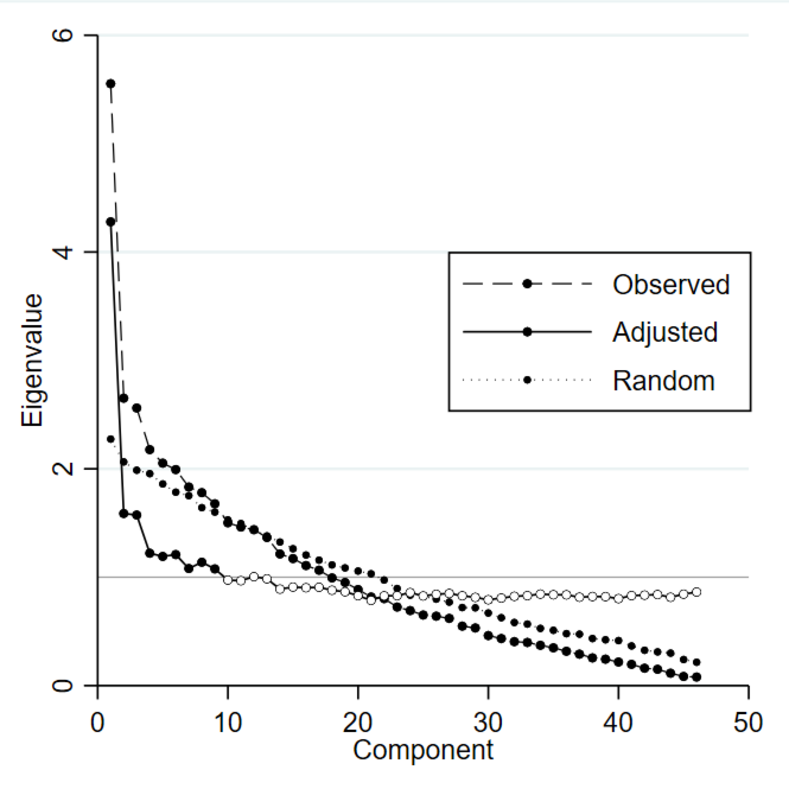

Supplement: S2 Fig — (TIF) [file pone.0298928.s002.tif]

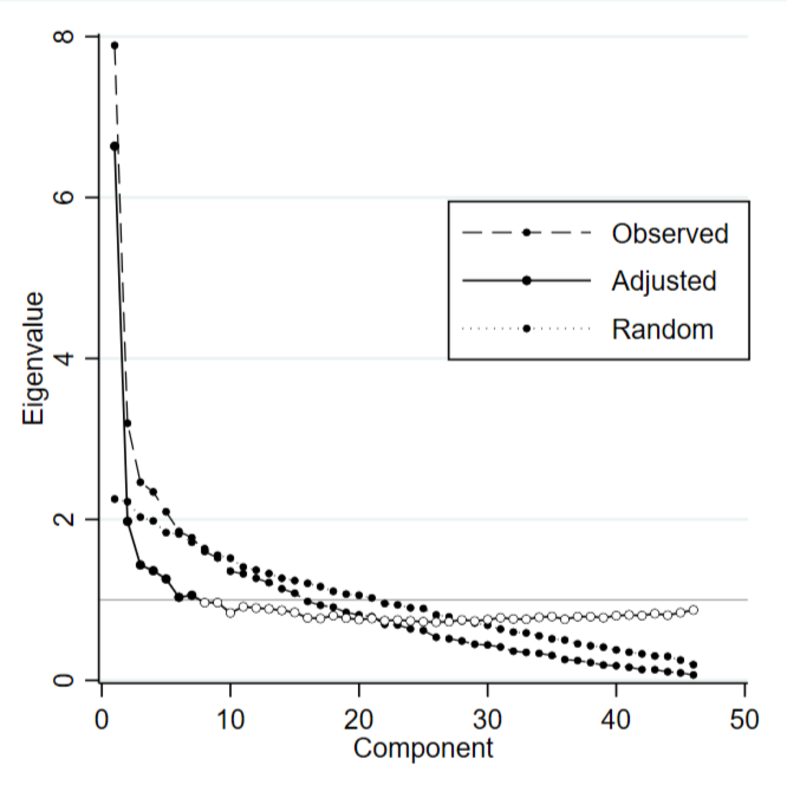

Supplement: S3 Fig — (TIF) [file pone.0298928.s003.tif]

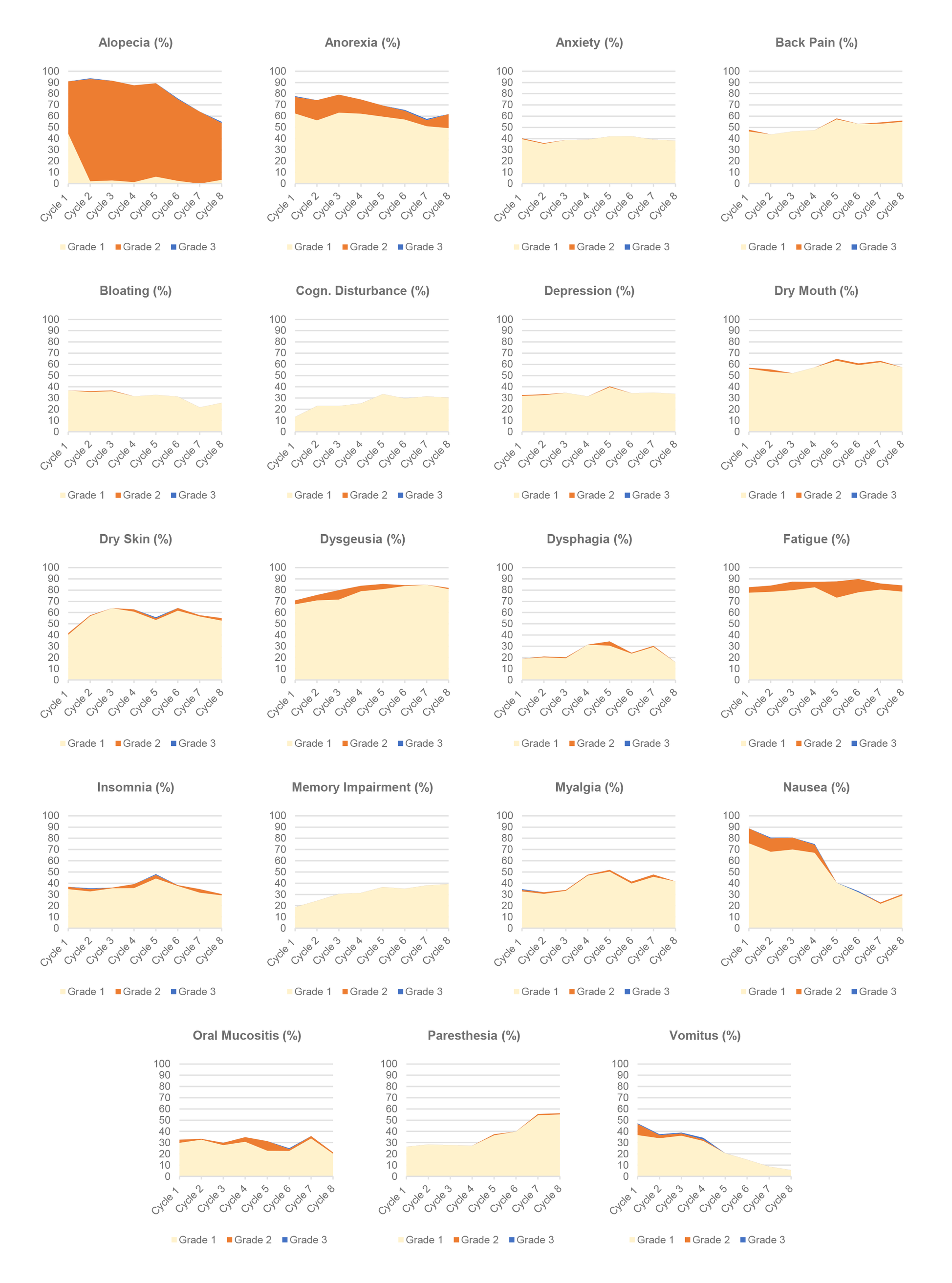

Supplement: S4 Fig — (TIF) [file pone.0298928.s004.tif]
