# Supplementary material for: Patient-reported outcomes and symptom clusters pattern of chemotherapy-induced toxicity in patients with early breast cancer
Source: PLoS One. 2024 Feb 23;19(2):e0298928. doi: 10.1371/journal.pone.0298928 (PMC10890761; doi:10.1371/journal.pone.0298928)
Supplement: S1 Table — (DOCX) [file pone.0298928.s005.docx]

| **S1 Table. Incidence of patient-reported chemotherapy toxicity symptoms based on severity grade throughout chemotherapy** | | | | |
| --- | --- | --- | --- | --- |
| **Toxicities** | **Incidence (%)** | **Grade 1 Toxicity (%)** | **Grade 2 Toxicity (%)** | **Grade 3 Toxicity (%)** |
| Dry mouth | 91.5 | 86.6 | 4.9 | 0 |
| Oral mucositis | 72.5 | 54.9 | 16.9 | 0.7 |
| Sore throat | 33.1 | 33.1 | 0 | 0 |
| Dysphagia | 66.2 | 60.6 | 5.6 | 0 |
| Dysgeusia | 97.2 | 79.6 | 17.6 | 0 |
| Anorexia | 97.9 | 57.0 | 38.7 | 2.1 |
| Nausea | 96.5 | 69.0 | 25.4 | 2.1 |
| Vomiting | 76.8 | 61.3 | 13.4 | 2.1 |
| Dyspepsia | 74.7 | 63.4 | 11.3 | 0 |
| Flatulence | 90.8 | 90.1 | 0.7 | 0 |
| Bloating | 73.2 | 71.8 | 1.4 | 0 |
| Constipation | 80.3 | 73.9 | 5.6 | 0.7 |
| Diarrhea | 58.5 | 47.9 | 7.7 | 2.8 |
| Abdominal pain | 63.4 | 57.0 | 6.3 | 0 |
| Dyspnea | 42.3 | 41.5 | 0.7 | 0 |
| Cough | 74.6 | 66.9 | 7.7 | 0 |
| Palpitation | 49.3 | 49.3 | 0 | 0 |
| Back pain | 84.5 | 81.0 | 3.5 | 0 |
| Paresthesia | 76.1 | 73.9 | 2.1 | 0 |
| CIPN | 65.5 | 63.4 | 2.1 | 0 |
| Myalgia | 83.8 | 76.1 | 7.0 | 0.7 |
| Arthralgia | 68.3 | 62.7 | 5.6 | 0 |
| Dry skin | 92.3 | 84.5 | 7.0 | 0.7 |
| Pruritus | 78.9 | 73.2 | 5.6 | 0 |
| Skin hyperpigmentation | 90.1 | 85.2 | 4.9 | 0 |
| Hyperhidrosis | 52.1 | 52.1 | 0 | 0 |
| Body odor | 59.9 | 59.9 | 0 | 0 |
| Headache | 87.3 | 79.6 | 7.7 | 0 |
| Dizziness | 90.1 | 80.3 | 9.8 | 0 |
| Cognitive disturbance | 55.6 | 55.6 | 0 | 0 |
| Memory impairment | 60.6 | 60.6 | 0 | 0 |
| Insomnia | 79.6 | 69.7 | 8.4 | 1.4 |
| Fatigue | 98.6 | 62.7 | 35.9 | 0 |
| Anxiety | 68.3 | 67.6 | 0.7 | 0 |
| Depression | 64.8 | 64.1 | 0.7 | 0 |
| Alopecia | 99.3 | 0 | 99.3 | 0 |
| Nail discoloration | 98.6 | 98.6 | 0 | 0 |
| Nail ridges | 93.7 | 93.7 | 0 | 0 |
| Watery eyes | 70.4 | 69.7 | 0.7 | 0 |
| Blurred vision | 64.8 | 62.7 | 2.1 | 0 |
| Tinnitus | 51.4 | 50.7 | 0.7 | 0 |
| Irregular menstruation | 45.1 | 18.3 | 22.5 | 4.2 |
| Vaginal discharge | 40.8 | 38.7 | 2.1 | 0 |
| Vaginal dryness | 43.0 | 38.7 | 4.2 | 0 |
| Decreased libido | 45.1 | 44.4 | 0.7 | 0 |
| Hot flushes | 61.3 | 60.6 | 0.7 | 0 |
| Total | 72.0 | 62.9 | 8.7 | 0.4 |
| Abbreviations: CIPN =chemotherapy-induced peripheral neuropathy. | | | | |
