# Supplementary material for: Patient-reported outcomes and symptom clusters pattern of chemotherapy-induced toxicity in patients with early breast cancer
Source: PLoS One. 2024 Feb 23;19(2):e0298928. doi: 10.1371/journal.pone.0298928 (PMC10890761; doi:10.1371/journal.pone.0298928)
Supplement: Attachment — Submitted filename: Response to Reviewers.docx [file pone.0298928.s007.docx]

**Response to Reviewers**

Dear Editor and Reviewer,

We appreciate the positive feedback provided by the editorial team and reviewers 1 and 2. We are grateful for the chance to address the constructive points raised in our revised manuscript. Below, you will find our detailed response to the feedback, highlighting the relevant changes we've implemented. Following the given instructions, we have uploaded both a clean copy and a track changes version of the revised manuscript, along with supplementary files as needed.

**Response to Editor’s comment**

1. Please ensure that your manuscript meets PLOS ONE's style requirements, including those for file naming.

**Response:**

We have adjusted the abstract section to comply with the 300-word limit. Additionally, we have verified other requirements before submitting our revised manuscript and ensured that it aligns with PLOS ONE's style guidelines.

2. In your Data Availability statement, you have not specified where the minimal data set underlying the results described in your manuscript can be found. PLOS defines a study's minimal data set as the underlying data used to reach the conclusions drawn in the manuscript and any additional data required to replicate the reported study findings in their entirety. All PLOS journals require that the minimal data set be made fully available. For more information about our data policy, please see http://journals.plos.org/plosone/s/data-availability.

Upon re-submitting your revised manuscript, please upload your study’s minimal underlying data set as either Supporting Information files or to a stable, public repository and include the relevant URLs, DOIs, or accession numbers within your revised cover letter. For a list of acceptable repositories, please see http://journals.plos.org/plosone/s/data-availability#loc-recommended-repositories. Any potentially identifying patient information must be fully anonymized.

Important: If there are ethical or legal restrictions to sharing your data publicly, please explain these restrictions in detail. Please see our guidelines for more information on what we consider unacceptable restrictions to publicly sharing data: http://journals.plos.org/plosone/s/data-availability#loc-unacceptable-data-access-restrictions. Note that it is not acceptable for the authors to be the sole named individuals responsible for ensuring data access.

We will update your Data Availability statement to reflect the information you provide in your cover letter.

**Response:**

We have specified in our data availability statement upon resubmitting that the minimal data set is available as a supporting information file.

**Response to Reviewer #1**

1. In the introduction, third paragraph the author refers to the CTCAE as emphasizing patient-reported toxicity. This is confusing as the CTCAE is not a patient reported measure and is completed by clinicians albeit as part of an assessment process hopefully in dialogue with the patient.

**Response:**

Thank you for your remarks. We have edited the third paragraph in the introduction accordingly.

2. Methods: The authors described developing a patient reported version in this study that was translated. It was not clear if best practices were followed for cultural and linguistic validation and if psychometric validity was assessed of the translated version or was this just a straight language translation. This raises questions about reliability and validity of what they are calling a “patient version” of the CTCAE. It was not entirely clear why they did not use the well-validated PRO-CTCAE. At a minimum there should be some reference to the PRO-CTCAE in the discussion and at a minimum the measure developed for this study should be addressed as a possible limitation and more detail provided about this measure and its validity, etc. Additionally, it is not clear of those approached for the study how many declined (no consort chart) as to determine if any selection bias was introduced into the study and the observational nature of the study should also be addressed in the limitation. Additionally, the PRO version appears to be interviewer administered and what method was used for this and the specifics of this need to be further outlined i.e. was a script followed, provision of any interpretation, etc.

**Response:**

Thank you for your kind remarks. The PRO-CTCAE was designed to be used as a companion to the CTCAE, which includes 124 items representing symptomatic toxicities drawn from the CTCAE. It was designed to allow patients to report their symptoms in conjunction with the CTCAE that were clinician-reported. At the time of study initiation, we decided to develop our patient-reported version of CTCAE and not adapt PRO-CTCAE mainly for two reasons.

First, there are no standardized scoring rules for combining attributes (severity and interference) into a single score in some of the PRO-CTCAE symptom items. For instance, in the assessment of oral mucositis, the CTCAE questionnaire provides grading definition for each grade level: 1=asymptomatic or mild symptoms; intervention not indicated; 2=Moderate pain; not interfering with oral intake; modified diet indicated; 3=Severe pain, interfering with oral intake; and 4=Life-threatening consequences, urgent intervention indicated. In PRO-CTCAE, the assessment of oral mucositis is further divided into severity and interference assessment. For severity, the options provided are none/mild/moderate/severe/very severe. For interference, the options provided are not at all/a little bit/somewhat/quite a bit/very much. The toxicity grade provided by CTCAE has combined attributes (severity and interference) compared to what is questioned in PRO-CTCAE.

Additionally, PRO-CTCAE was ideally implemented through a web-based or software platform, or a telephone interactive voice response system, allowing patients to independently report their symptoms. However, In our study population, many of our patients were not technologically illiterate, and we currently lack resources to facilitate electronic questionnaire administration. Despite providing patients with a paper form of the questionnaire to take home and complete, the diverse educational backgrounds of the patients posed challenges in effectively filling out the questionnaire. During the interview session, the clinician's interpretation of the symptoms data was not incorporated, and the interviewer only helped patients report their symptoms, making it a patient-centered reporting.

We have also added some references on PRO-CTCAE in the discussion section accordingly.

In our revised manuscript, additional information has been incorporated concerning the developmental stages of the questionnaire. Specifically, we undertook face validity evaluations involving both experts and potential respondents. Prior to the deployment of the questionnaire, we also conducted pilot testing to refine the questionnaire further. Notably, our study has yet to undergo a comprehensive psychometric assessment encompassing validity and reliability evaluations, a limitation acknowledged in our study and added in the revised manuscript. Furthermore, the revised manuscript has included details regarding the interviewer's approach to questionnaire delivery.

Concerning the recruitment of participants for this study, our utilization of consecutive sampling methods has allowed for the inclusion of all breast cancer patients visiting our oncology center during the inclusion period. While it is acknowledged that certain individuals declined participation, comprehensive records detailing the total number of potential subjects approached and the corresponding number declining to participate in the study were not maintained. In the revised manuscript, we have acknowledged this limitation, related to this study's observational design, which may introduce selection bias and limit the generalizability of our findings.

3. The author states in the fourth paragraph in the introduction that there are many studies of symptom clusters but no references are provided, the review papers should be referenced.

**Response:**

Thank you for your remarks. We have added the reference in the revised manuscript accordingly.

4. The adequacy of using a factor analysis approach and why this methodology was used. See Helen Skerman paper on comparison of factor analysis methods and accuracy in identification of symptom clusters. J of Pain and Symptom Management 2012 and there are papers criticising this as a limited approach and new methods recommended. Please address this as to justify this approach and the potential limitations; and how rigor in cluster identification was assured.

**Response:**

Thank you for your remarks. We have elaborated upon the rationale for selecting the factor analysis method in our revised manuscript. Additionally, we have acknowledged and addressed potential limitations associated with the chosen factor analysis method.

**Response to Reviewer #2**

1. CTCAE does not emphasize patient-reported outcomes. The interview process to assesses adverse events described in the Methods section is based on the interviewer interpretation of the record of patient reporting their adverse events. The adverse events are not self-reported, therefore the patients experience is still subject to observer bias. In the recent years, there is an extensive literature discussing Patient Reported Outcome CTCAE. Please see below:

Basch E, Reeve BB, Mitchell SA, Clauser SB, Minasian LM, Dueck AC, Mendoza TR, Hay J, Atkinson TM, Abernethy AP, Bruner DW. Development of the National Cancer Institute’s patient-reported outcomes version of the common terminology criteria for adverse events (PRO-CTCAE). Journal of the National Cancer Institute. 2014 Sep 1;106(9):dju244.

Instead, authors should emphasize that CTCAE has become standard in cancer clinical trials and their application outside of the context of a clinical trial in a prospective data collection is somewhat novel.

**Response:**

Thank you for your kind remarks. The evaluation of chemotherapy toxicity does not rely on the interviewer interpretation of patient-recorded data. Instead, it is derived directly from patients' responses during interviews, with no clinician or interviewer interpretations incorporated into the data. The revised manuscript now provides additional information on the interviewer's approach to questionnaire delivery.

In the revised manuscript, we have incorporated the rationale behind the development of our novel approach involving the adaptation of the CTCAE questionnaire (commonly utilized in clinical trial settings) into a patient-reported format, coupled with an interview process.

While acknowledging the existence of the Patient-Reported Outcomes version of the Common Terminology Criteria for Adverse Events (PRO-CTCAE), designed for self-reporting of symptomatic toxicities, we highlight several challenges in its implementation in our study.

First, certain symptom items in PRO-CTCAE have a dual assessment for severity and interference, and there are currently no standardized scoring rules for merging these attributes into a unified score in a single symptom.

Moreover, ideally, the PRO-CTCAE questionnaire is implemented through a web-based software platform or a telephone interactive voice response system, allowing patients to report their symptoms independently. However, in our study population, many of our patients were not technologically illiterate, and we currently lack the resources to facilitate electronic questionnaire administration. Despite providing patients with a paper form of the questionnaire to take home and complete, the diverse educational backgrounds of the patients posed challenges in effectively filling out the questionnaire.

2. Please use the terms dichotomize or categorize instead of divide when describing variables under Methods

**Response:**

Thank you for your remarks. We have changed the terms accordingly.

3. Please provide more details about the interview process to assess adverse events. Were patients provided with the list of 46 pre-selected AEs? Did the interview ask specific questions? It is unclear whether the adverse events were self-reported by patients or interpreted by the interviewers.

**Response:**

Thank you for your remarks. The patients were not provided with checklist of 46 pre-selected toxicity types. From the 80 symptoms in the questionnaire, we extracted 46 symptoms of chemotherapy side effect, which were selected a priori.

Instead, during the interview, patients were first asked to report symptoms occurrence in the past week. For each symptom occurrence mentioned, details regarding the grade based on the CTCAE definition were further asked, with each definition explained by the interviewer. After all symptoms were mentioned, the interviewer was to ask about the occurrence and grade of all other symptoms not previously mentioned by the subject, following the list of symptoms in the script and first mentioning the definition of each symptom asked. All answers from the subject were recorded on the interviewee script’s checklist and then were later input into the database.

We have added the details regarding the interview process in the revised manuscript accordingly.

4. The term bivariate linear regression is not adequate. In the context of regression models, there are univariable (one predictor/independent variable) and multivariable (two or more predictors/independent variables). In the context of test of hypotheses, the term bivariate analysis is commonly used but not for regression models.

**Response:**

Thank you for your remarks. We have changed the terms accordingly.

5. The number of adverse events is counting data. Is it the normality assumption valid for the fitted regression models? Generalized linear models such as Poisson and negative binomial seem more appropriate. Nowadays, they are standard analysis. Is there a reason that those methods were not considered?

**Response:**

Thank you for your comments. We have undertaken a reanalysis, employing Poisson regression for the statistical analysis.

6. Which type of data was used as input for EFA? Presence/absence of AE or grade of AE? EFA assumes that data is normally distributed. That does not seem the case. Please consider methods that are appropriate for categorical data such as correspondence analysis. Please see references below:

Sourial N, Wolfson C, Zhu B, Quail J, Fletcher J, Karunananthan S, Bandeen-Roche K, Béland F, Bergman H. Correspondence analysis is a useful tool to uncover the relationships among categorical variables. Journal of clinical epidemiology. 2010 Jun 1;63(6):638-46.

Diniz MA, Gresham G, Kim S, Luu M, Henry NL, Tighiouart M, Yothers G, Ganz PA, Rogatko A. Visualizing adverse events in clinical trials using correspondence analysis with R-package visae. BMC medical research methodology. 2021 Dec;21:1-1.

**Response:**

Thank you for your remarks. The type of data used in EFA is the occurrence of toxicity.

EFA conventionally assumes multivariate normality. In adapting to the presence of binary variables in our EFA, we opted for tetrachoric correlations instead of Pearson correlations to construct the association matrix, which is commonly employed when handling binary variables [1-2]. This approach also aligns with methodologies employed in prior EFA studies utilizing binary variables [3-6].

We appreciate your suggestion to incorporate correspondence analysis. However, at present, we still need more proficiency and experience in employing this method. We are open to exploring this method in our future studies.

[1] Schreiber, James B. "Issues and recommendations for exploratory factor analysis and principal component analysis." Research in Social and Administrative Pharmacy 17.5 (2021): 1004-1011.

[2] Kubinger, Klaus D. "On artificial results due to using factor analysis for dichotomous variables." Psychology science 45.1 (2003): 106-110.

[3] Harris, Carolyn S., et al. "Symptom clusters in patients receiving chemotherapy: a systematic review." BMJ Supportive & Palliative Care 12.1 (2022): 10-21.

[4] Mazor, Melissa, et al. "Differences in symptom clusters before and twelve months after breast cancer surgery." European Journal of Oncology Nursing 32 (2018): 63-72.

[5] Sullivan, Carmen W., et al. "Stability of symptom clusters in patients with breast cancer receiving chemotherapy." Journal of pain and symptom management 55.1 (2018): 39-55.

[6] Sullivan, Carmen Ward, et al. "Differences in symptom clusters identified using symptom occurrence rates versus severity ratings in patients with breast cancer undergoing chemotherapy." European Journal of Oncology Nursing 28 (2017): 122-132.

7. Were the three treatment regimens considered in the same EFA analysis? Would the cluster be confounded by treatment?

**Response:**

All three treatment categories were included in the EFA analysis. Principal-component factoring (PCF, or principal component analysis/PCA) was utilized in our EFA. This method ensures that the analysis remains unaffected by variations in chemotherapy regimens, as PCF disregards individual-specific factors and unique variance (in this case, different chemotherapy regimens).

Given the extensive number of toxicity variables, our primary objective is data reduction and discovering general patterns in our local population. We intend to focus on the minimum number of factor solutions needed to encompass the maximum proportion of the total variance in the original set of variables. Consequently, we have chosen to employ the PCF method. We have also added this explanation to the revised manuscript.

8. Were p-values in Table 2 and 5 corrected for multiple comparisons such as Holm or FDR? Otherwise, a large number of false positive is expected.

**Response:**

Thank you for your remarks. While we recognize the importance of addressing the issue of multiple comparisons, we still need to improve our proficiency in applying Holm or FDR correction statistical analysis. We remain open to further guidance and suggestions for addressing this concern.

As our study is exploratory, we aim to discover potential unique relationships and patterns between the independent variable and each outcome variable separately. While reducing the risk of Type I errors, these corrections increase the risk of Type II errors and may hinder potentially significant findings. We are imperatively inclined to avoid the type II error. As In Table 2, the harmful effect that chemotherapy toxicity brings on patients' overall treatment experience and functional status based on chemotherapy regimen might be significant for clinical practice. As in the possible correlation between symptom clusters in Table 5, association findings gave an interesting insight for future toxicity symptom cluster exploration and intervention.

We have acknowledged this limitation in the revised manuscript and encourage prospective studies to employ confirmatory analyses with stringent multiple comparison controls to validate noteworthy findings from our exploratory investigation.

Following our revisions, we hope the manuscript is suitable for publication in PLOS ONE.

Yours sincerely,

Susanna Hutajulu, MD, PhD
